# From imaging heterogeneity to clinical decision: a novel imaging biomarker based on bone marrow kinetics for advanced NSCLC patients in partial response

**DOI:** 10.1186/s13550-026-01440-w

**Published:** 2026-05-21

**Authors:** Yubo Wang, Zhiheng Yao, Jingjing Wan, Yuan Cai, Xinyu Yang, Rongliang Wu, Kebir Sied, Tao Sun, Ying Liang

**Affiliations:** 1 Department of Nuclear Medicine, National Cancer Center, National Clinical Research Center for Cancer/Cancer Hospital & Shenzhen Hospital, Chinese Academy of Medical Sciences and Peking Union Medical College, Shenzhen, China; 2 Lauterbur Research Center for Biomedical Imaging, Shenzhen Institute of Advanced Technology, Chinese Academy of Sciences, Shenzhen, China; 3 Division of Clinical Neuro-Oncology, Department of Neurology and Center for Translational Neuro- and Behavioral Sciences (C-TNBS), University Hospital Essen, University of Duisburg-Essen, 45147 Essen, Germany; 4 Department of Nuclear Medicine, The Fifth Affiliated Hospital, Sun Yat-sen University, Zhuhai, 519000 Guangdong Province China; 5 GCP-compliant Phase I Clinical Trial Ward, Shenzhen Third People’s Hospital, The Second Affiliated Hospital of Southern, University of Science and Technology, Shenzhen, Guangdong China

**Keywords:** Non-small cell lung cancer, Bone marrow, PET/CT, Treatment response

## Abstract

**Background:**

Risk of disease progression varies substantially among advanced non-small cell lung cancer (NSCLC) patients who achieve a partial response to initial therapy, and this subgroup currently lacks practical prognostic tools. This study aimed to develop a novel imaging biomarker by assessing bone marrow kinetic heterogeneity via pretreatment dynamic ^18^F-fluorodeoxyglucose positron emission tomography/computed tomography (^18^F-FDG PET/CT). We retrospectively analyzed 51 patients with advanced NSCLC, focusing on the 32 who attained a partial response following first-line treatment. Time-activity curves (TACs) from the tumor and bone marrow were metabolically decomposed. Through integrated analysis of clinical characteristics, conventional PET parameters, and 28 TAC-derived features, coupled with a rigorous bootstrap-LASSO-Cox procedure (1000 resamples) for robust feature selection, we developed a multivariate Cox model and a simplified risk-stratification model.

**Results:**

The bootstrap-LASSO-Cox analysis identified the slope of the bone marrow free FDG TAC between 10 and 30 min post-injection (BM_C_f__Slope_10−30_) as the most significant independent predictor. In the multivariate Cox model, this feature was strongly associated with a reduced risk of progression (Hazard Ratio = 0.10, 95% CI: 0.03–0.35, *P* < 0.001) and demonstrated good discriminative ability (C-index = 0.77, 95% CI: 0.68–0.86). Using an optimal cutoff value of -0.01119, this single parameter significantly stratified patients into high- and low-risk groups with distinct progression-free survival (Log-rank *P* < 0.001). The simplified model showed robust predictive performance, with average time-dependent AUCs of 0.792, 0.774, and 0.731 at 12, 18, and 24 months, respectively, and provided clinical net benefit. Neither conventional metabolic parameters nor clinical characteristics showed significant predictive value.

**Conclusion:**

For advanced NSCLC patients achieving an initial partial response, pretreatment assessment of bone marrow kinetics via dynamic ^18^F-FDG PET/CT emerges as a potential prognostic biomarker. The TAC slope feature, which reflects early interstitial clearance, can identify patients at high risk of early progression, offering potential for personalizing surveillance strategies and guiding timely therapeutic interventions.

**Supplementary Information:**

The online version contains supplementary material available at 10.1186/s13550-026-01440-w.

## Introduction

The management of advanced non-small cell lung cancer (NSCLC) has been revolutionized by immunotherapy and targeted therapies, leading to significantly improved objective response rates. Consequently, a substantial proportion of patients achieve partial response (PR) following first-line treatment, marking a pivotal therapeutic milestone [1]. However, this success reveals a critical clinical dilemma: the PR category encompasses a profoundly heterogeneous population with divergent outcomes. While some patients enjoy sustained disease control, a concerning subset, often within 12 months, experiences early progression. This leads to renewed morbidity, psychological distress, and compromised quality of life [2]. Such unpredictability renders the conventional PR designation insufficient for guiding subsequent clinical decisions, creating an urgent need for biomarkers that can effectively substratify risk within this specific patient group.

Current prognostic tools are limited in meeting this challenge. Clinicopathological variables, such as performance status and PD-L1 expression, alongside static ^18^F-FDG PET/CT parameters like the maximum standardized uptake value (SUVmax) and metabolic tumor volume (MTV), lack the sensitivity to capture the underlying biological drivers of early progression in patients who have achieved PR [3, 4]. Crucially, by focusing predominantly on tumor-centric measures, these tools fail to assess the dynamic, systemic host response to residual disease. This response is likely a decisive factor in determining the durability of a partial response. Therefore, identifying novel, mechanistically informed biomarkers is imperative to enable personalized surveillance and timely therapeutic intervention for this clinically ambiguous yet sizable subgroup.

^18^F-FDG PET/CT serves as a cornerstone of functional imaging in oncology [5–7]. However, standard static protocols offer only a snapshot of metabolic activity, overlooking the dynamic kinetic processes of tracer uptake. In contrast, dynamic PET imaging acquires sequential data to generate time-activity curves (TACs), which quantify the temporal evolution of ^18^F-FDG metabolism [8]. This approach provides superior sensitivity to physiological heterogeneity and early pathophysiological shifts [9]. Despite this potential, the use of dynamic PET biomarkers has been largely confined to describing baseline tumor biology. However, their utility for prognosticating long-term outcomes in responding patients has not been established. Specifically, the value of pre-treatment scans for risk stratification within the future partial response cohort remains unexplored. This gap is especially prominent for metrics derived from the systemic bone marrow microenvironment.

The bone marrow is increasingly recognized as a pivotal mediator of tumor progression and treatment response [10]. Beyond its hematopoietic role, it functions as a critical immunomodulatory niche, supplying myeloid and immune cells that can either suppress or support tumor growth [11]. Tumor-derived signals can pathologically remodel this niche, inducing metabolic reprogramming, stromal dysfunction, and immunosuppression. These alterations may fuel early disease progression despite an initial radiographic response [12, 13]. Importantly, the degree of bone marrow dysregulation is likely heterogeneous across patients, potentially mirroring the variable host responses to residual disease after therapy.

For these reasons, our study focuses specifically on advanced NSCLC patients with PR, examining how bone marrow glucose kinetics influence their outcomes. This group represents a critical decision point where choices regarding therapy continuation, modification, or intensification are poorly informed by existing tools. We hypothesize that dynamic ^18^F-FDG PET-derived features of bone marrow kinetic heterogeneity reflect this variable host response and serve as a superior predictor of progression-free survival compared to conventional static or clinical parameters. This study aims to: (1) quantify bone marrow kinetic heterogeneity via dynamic PET time-activity curve analysis; (2) validate its independent prognostic value against established risk factors; and (3) develop and validate an integrated clinical risk-stratification model to guide personalized management for this underserved population.

## Materials and methods

### Patients

**Fig. 1:**
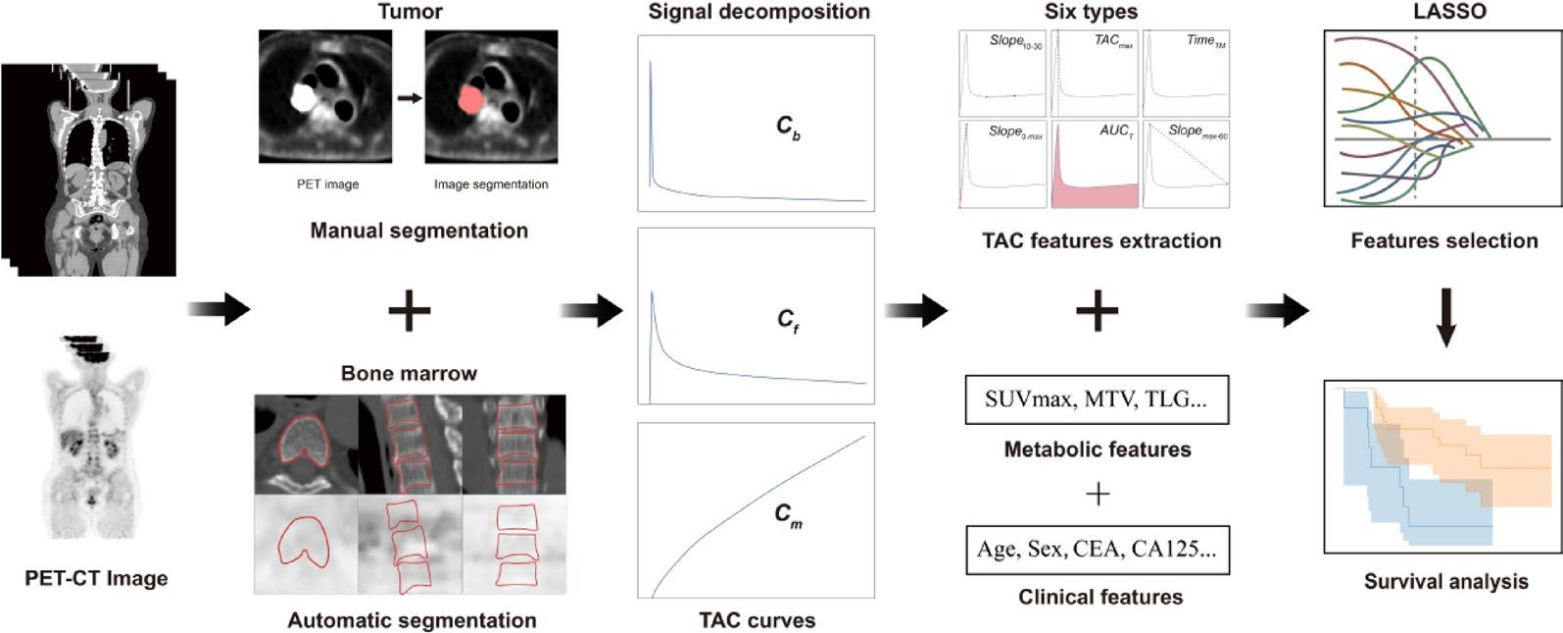
Overview of the workflow for feature extraction from dynamic PET imaging. PET-CT images were used to segment the tumor manually and the bone marrow automatically. TACs were obtained from these regions and decomposed into three signal components: blood component ($\:{C}_{b}$), free component ($\:{C}_{f}$), and metabolism component ($\:{C}_{m}$). Six types of features were extracted directly from these decomposed signals. Feature selection was then performed using LASSO regression to identify the most significant TAC features for analysis

This retrospective observational cohort study was conducted with the approval of the Institutional Ethics Committee (Approval No. KYLH2022-1) and in accordance with the Declaration of Helsinki. The study is reported in accordance with the STROBE (Strengthening the Reporting of Observational Studies in Epidemiology) guidelines. A completed STROBE checklist is provided as Supplementary File 1. The primary analytical aim was to perform a conditional prognostic analysis within the subgroup of patients who achieved an early partial response, to investigate whether baseline characteristics could stratify subsequent outcomes among these initial responders. Data were extracted from the hospital’s electronic medical records for eligible patients treated between November 2022 and January 2024. While written informed consent had been obtained for the original PET/CT scans, the ethics committee granted a waiver for specific consent for this retrospective analysis. All data were anonymized prior to analysis. The study design is summarized in Fig. 1.

A total of 51 consecutive patients with pathologically confirmed advanced NSCLC were included in this retrospective cohort. Advanced NSCLC was defined as stage IIIB–IV disease according to the American Joint Committee on Cancer (AJCC) 8th edition TNM staging system. Clinical staging was determined at initial diagnosis by a multidisciplinary tumor board. All dynamic ^18^F-FDG PET/CT scans were performed pre-treatment, with a mandated interval of ≤ 14 days prior to the initiation of first-line systemic therapy. This pre-treatment imaging protocol was specifically designed to ensure that the observed bone marrow metabolic activity reflected the disease state itself, prior to and independent of any potential confounding effects from systemic anticancer therapies.

Inclusion criteria were: (1) histologically confirmed advanced NSCLC; (2) evaluable pre-treatment dynamic ^18^F-FDG PET/CT with all required frames after quality control; and (3) complete pre-specified clinical covariates and treatment data per NCCN guidelines (Version 2.2022). Exclusions for metabolic confounders: To minimize confounding effects on bone marrow glucose metabolism, patients with active infection, recent use (within 4 weeks) of granulocyte colony-stimulating factor, or concurrent corticosteroid therapy at the time of scanning were excluded from the primary analysis. No patients met these criteria (*N* = 0). Exclusions to define the analytical cohort: (1) absence of a RECIST 1.1-defined partial response at the first post-baseline imaging assessment (typically 9 to 12 weeks after treatment initiation), as the present study aimed to specifically investigate predictors of durable response among initial responders (*N* = 16); and (2) lack of sufficient radiographic follow-up (a priori defined as ≥ 12 months or until a documented progression event) to determine progression-free survival status (*N* = 3).

A total of 32 patients who met al.l eligibility criteria were included in the final analysis. Tumor response and progression were assessed in accordance with RECIST 1.1 criteria. All imaging studies were centrally reviewed by two independent, blinded radiologists (YW, YL). For this analysis, disease progression was strictly defined as progressive disease (PD) confirmed by RECIST 1.1 [14]. Progression-free survival (PFS), defined as the time from treatment initiation to either PD or death from any cause, was calculated with censoring applied at the last radiographic follow-up.

### Dynamic PET/CT imaging

All participants fasted for a minimum of 6 h prior to imaging. Venous plasma glucose was measured to ensure a level below 11.1 mmol/L (200 mg/dL) prior to injection. Following intravenous administration of 18F-FDG (mean activity 3.5 MBq/kg, range 3.1 to 4.0 MBq/kg; injected as a slow bolus over approximately 30 s), patients remained immobile in the supine position for a standardized uptake period of 60 ± 5 min before the start of the dynamic acquisition. Imaging was performed on a Discovery MI PET/CT scanner (GE Healthcare). The protocol commenced with a low-dose CT scan (120 kV; tube current 180 to 350 mA; pitch 1.375:1; noise index 13) from the head to mid-femur for attenuation correction and anatomical localization. Immediately thereafter, a dynamic list-mode PET acquisition of the chest was initiated and continued for 65 min, divided into 28 frames (6 × 10 s, 4 × 30 s, 4 × 60 s, 4 × 120 s, 10 × 300 s). A subsequent static whole-body PET scan was also acquired. PET images were reconstructed using the Block Sequential Regularized Expectation Maximization algorithm with a β-value of 350, 3 iterations, 16 subsets, a 256 × 256 matrix, and a voxel size of 2.73 × 2.73 × 2.78 mm³. Reconstruction incorporated time-of-flight and point-spread-function modeling, along with all standard corrections (attenuation, scatter, randoms, dead time, and decay). Standardized uptake values were normalized to body weight. This protocol adhered to relevant international guidelines for ^18^F-FDG PET/CT tumor imaging [15].

### Tumor ROI manual delineation

Tumor regions of interest (ROIs) were manually segmented on PET images using ITK-SNAP software (Version 4.0.2). All initial delineations were performed by one radiologist (YW, 6 years of PET experience). To evaluate inter‑observer reproducibility, a second radiologist (JW, 11 years of PET experience) independently re‑segmented a randomly selected subset of 15 cases. A standardized protocol was followed in which tumor boundaries were delineated slice‑by‑slice using a fixed SUV threshold of 2.5, guided by the co‑registered CT images to exclude necrotic regions and adjacent physiologic uptake. In patients with multiple lesions, only the largest primary tumor was analyzed. Agreement between readers was quantified with the Dice similarity coefficient, which demonstrated excellent reproducibility (median Dice = 0.89, IQR: 0.85 to 0.92). Any discrepancies in the reproducibility subset were resolved through consensus discussion, and the same consensus principle was applied to finalize all segmentations.

### Bone marrow ROI automatic segmentation

Bone marrow ROIs were automatically generated from the low-dose CT images using TotalSegmentator (v2.1.0) [16]. The initial vertebral body segmentations were uniformly refined by applying a 3D morphological erosion with a spherical kernel of 1.5 mm radius to exclude the cortical bone and ensure analysis targeted the metabolically active marrow compartment. To restrict analysis to marrow unaffected by local disease or therapy, a systematic vertebral selection was performed by a radiologist (YW, 6 years of experience) using fused PET/CT images. A vertebra was excluded if it contained a CT-defined lytic/sclerotic lesion with corresponding focal PET hypermetabolism (indicative of metastasis). Starting from T3, the analysis descended caudally until a set of three consecutive, evaluable vertebrae within the T3-L1 range was identified. This principle of selecting specific, reproducible vertebral levels for bone marrow PET analysis is supported by established methodology in the field [17–19]. For dynamic PET analysis, each frame was first rigidly registered to the CT using a mutual information algorithm. To correct for potential patient motion during the 65-minute acquisition, all PET frames were then rigidly aligned to a common reference frame (a mid-timepoint, high-count frame). The CT-derived marrow ROIs were subsequently mapped onto this motion-corrected dynamic PET dataset for final quantification. All registration results underwent visual verification.

### Conventional metabolic features

Conventional PET features were quantified on the vendor workstation (GE AW VolumeShare 7) using the Q.Vol module. Standardized uptake values (SUVmax, SUVmin, and partial-volume corrected SUVmean) were normalized to the patient’s body weight. Metabolic tumor volume (MTV) was defined as the total volume of voxels with SUV ≥ 2.5, applying a fixed threshold commonly used in oncologic PET based on PERCIST-related literature [20]. Total lesion glycolysis (TLG) was computed as MTV × SUVmean.

### TAC Calculation

TACs were extracted for each tumor and bone marrow ROI as the mean activity concentration (kBq/mL) per dynamic frame, following a standard framework for dynamic PET analysis [8]. The frame timing followed the dynamic acquisition protocol described in the Imaging Protocol section above. For a given ROI encompassing $\:N$ voxels, the mean tissue activity concentration, denoted as $\:{C}_{T}\left(t\right)$, was calculated at each time frame *t* according to Eq. 1:

1 $$\:{C}_{T}\left(t\right)=\frac{1}{N}{\sum\:}_{i=1}^{N}{C}_{i}\left(t\right)$$

where $\:{C}_{i}\left(t\right)$ is the activity concentration in voxel $\:i$ at time 𝑡.

**Fig. 2:**
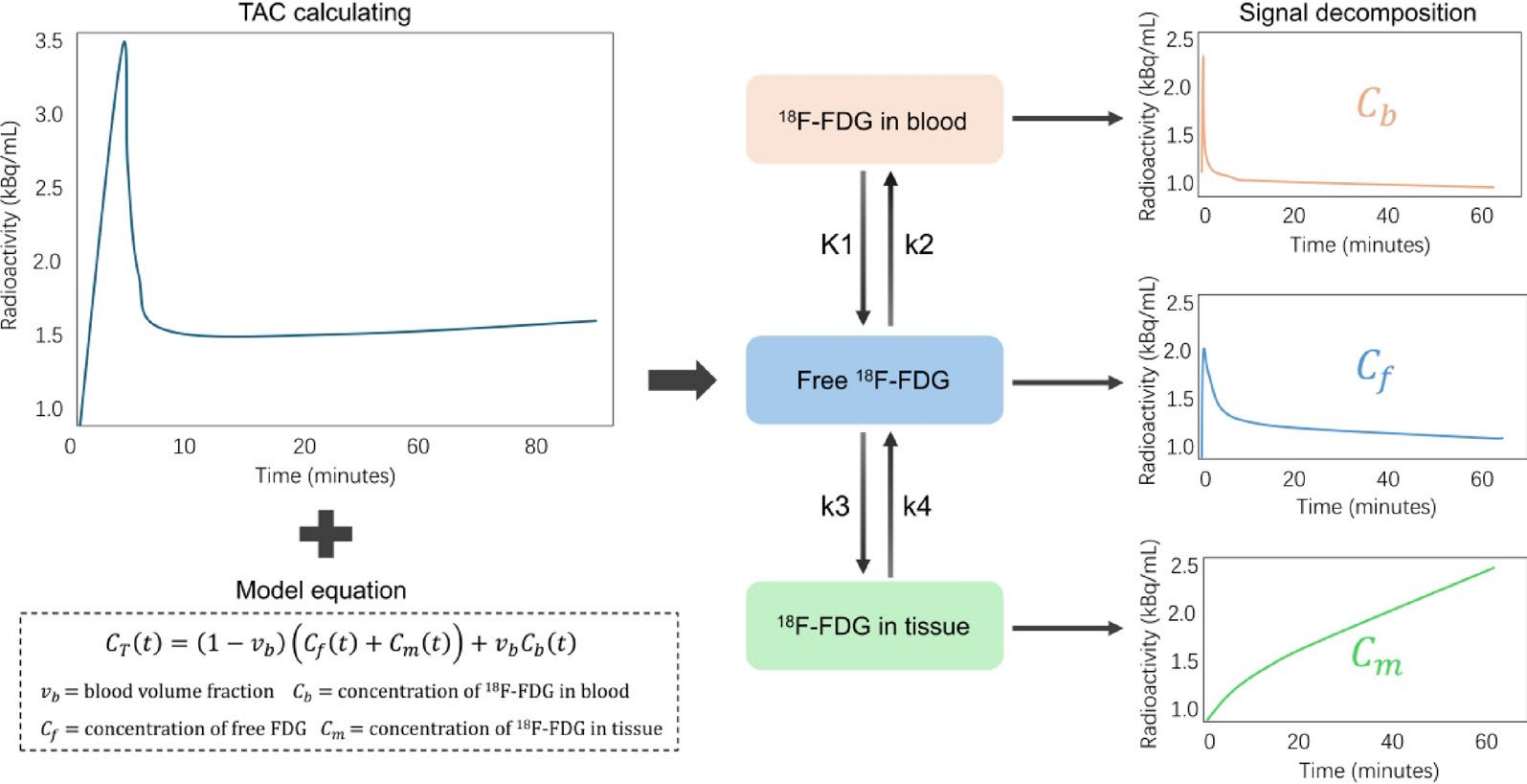
TAC compartmental signal decomposition for dynamic PET. Left, the measured tissue TAC is plotted over time (minutes). Center, a classical two-tissue model links ^18^F-FDG in blood, free ^18^F-FDG in tissue, and phosphorylated ^18^F-FDG in tissue, with transfer rate constants K₁ (blood to tissue), k₂ (tissue to blood), k₃ (phosphorylation), and k₄ (dephosphorylation). Right, model-derived component curves are shown for the vascular term $\:{C}_{b}$, free tissue term $\:{C}_{f}$, and metabolic term $\:{C}_{m}$. The modeled tissue concentration is given by Eq. 2, where $\:{v}_{b}$ is the blood volume fraction

To decompose the measured $\:{C}_{T}\left(t\right)$ into its underlying physiological components, a kinetic analysis was performed using the standard two-tissue model for ^18^F-FDG kinetics [21]. This model decomposes the measured tissue radioactivity signal into three physiological components, a process which is schematically illustrated in Fig. 2. Specifically, the model describes the total tissue activity$\:\:{C}_{T}\left(t\right)\:$as a combination of activity in the vascular space $\:{v}_{b}{C}_{b}\left(t\right)$, and the free $\:{C}_{f}\left(t\right)$ and metabolically trapped $\:{C}_{m}\left(t\right)$ compartments within the tissue:

2 $$\:{C}_{T}\left(t\right)=\left(1-{v}_{b}\right)\left({C}_{f}\left(t\right)+{C}_{m}\left(t\right)\right)+{v}_{b}{C}_{b}\left(t\right)$$

An image-derived input function for $\:{C}_{b}\left(t\right)$ was generated from the descending aorta by placing a 1-cm³ spherical ROI, and the subsequent estimation of model parameters were all conducted according to our prior work [22].

### TAC feature extraction

Based on the computed TAC ($\:{C}_{b}$, $\:{C}_{f}$ and $\:{C}_{m}$) for the target ROIs, six types of features were extracted to characterize the radiotracer dynamics: (1) $\:Slop{e}_{10-30}$: the slope of the TAC curve between 10 and 30 min, as defined in Eq. 3; (2) $\:{AUC}_{T}$: the cumulative radiotracer uptake over the entire temporal frame, calculated using Eq. 4; (3) $\:Slop{e}_{0-max}$: the slope of the TAC curve from the start to its peak, as defined in Eq. 5; (4) $\:Slop{e}_{max-60}$: the slope of the TAC curve from its peak to 60 min, as defined in Eq. 6; (5) $\:{Time}_{TM}$: the temporal point at which the TAC curve reaches its peak; (6) $\:{TAC}_{max}$: the peak value of the TAC curve. For $\:{C}_{m}$, due to its stable growth pattern (Fig. 1), we chose to extract two features: $\:Slop{e}_{0-max}$ and $\:{TAC}_{max}$. Finally, a total of 14 features were calculated for each ROI: 6 for $\:{C}_{f}$, 6 for $\:{C}_{b}$, and 2 for $\:{C}_{m}$.

3 $$\:Slop{e}_{10-30}=\frac{TAC\left(10\right)-TAC\left(30\right)}{20}$$

4 $$\:{AUC}_{T}={\sum\:}_{i=1}^{n-1}\frac{C\left({t}_{i}\right)+C\left({t}_{i+1}\right)}{2}\times\:\left({t}_{i+1}-{t}_{i}\right)$$

5 $$\:Slop{e}_{0-max}=\frac{TAC\left(max\right)-TAC\left(0\right)}{interval\_time}$$

6 $$\:Slop{e}_{max-60}=\frac{TAC\left(max\right)-TAC\left(60\right)}{interval\_time}$$

### Feature selection and model construction

We employed a bootstrap-LASSO-Cox approach for feature selection and model building. The detailed procedure is as follows. First, 1000 bootstrap resamples were drawn from the original dataset. For each resample, feature selection was performed using LASSO-Cox regression with 5-fold cross-validation and the 1-SE rule for λ. Features selected with a frequency greater than 50% across all resamples were identified as stable predictors. These stable features were subsequently incorporated into a multivariate Cox proportional hazards model. The discriminative ability of the model was quantified using the optimism-corrected Harrell’s C-index, estimated via bootstrapping (1000 repetitions).

### Development and evaluation of a simplified prognostic tool

To enhance clinical applicability, a simplified prognostic model was developed. From the stable feature pool, the most robust feature in the multivariate Cox analysis was chosen for risk stratification. The optimal cut-off for this feature was identified using the Log-rank test, dichotomizing patients into high- and low-risk groups. Survival differences between these groups were visualized with Kaplan-Meier curves and assessed statistically with the Log-rank test.

Model performance was comprehensively evaluated. Discriminative ability over time was assessed using time-dependent receiver operating characteristic (ROC) analysis based on Heagerty’s cumulative method [23], which calculated the Area Under the Curve (AUC) at 12, 18, and 24 months. Time-specific calibration performance, reflecting the agreement between predicted and observed survival probabilities, was evaluated at 12 months using a calibration curve that was validated internally with 1000 bootstrap resamples. The calibration slope and intercept were estimated to quantify the agreement. Furthermore, the clinical utility of the model was quantified at 12 months using decision curve analysis (DCA) for survival data to determine the net benefit across a range of clinically relevant risk thresholds.

### Statistical analysis

All statistical analyses were performed using Python (version 3.8). The specific packages and their versions used for analysis included pandas (v1.3.5), numpy (v1.24.4), scipy (v1.7.3), lifelines (v0.27.8), and statsmodels (v0.13.2). Continuous variables were assessed for normality using the Shapiro-Wilk test. Based on this assessment, two-sided independent t-tests were applied for normally distributed data; otherwise, the two-sided Mann-Whitney U test was used. Categorical variables were compared using Fisher’s exact test. A two-sided P value of less than 0.05 was considered statistically significant. The proportional hazards assumption for the Cox proportional hazards models was assessed using statistical tests based on Schoenfeld residuals. The proportional hazards assumption for the Cox proportional hazards models was assessed using statistical tests based on Schoenfeld residuals (implemented via the check_assumptions method in the lifelines package). No significant violations of the assumption were detected for the final prognostic model (global test *P* = 0.47), supporting the validity of the reported hazard ratios.

## Results

### Clinical and conventional metabolic features

Table 1 summarizes the baseline clinical and pathological characteristics of the progression (*N* = 17) and non-progression (*N* = 15) groups. The distribution of key demographic and clinical features, including age, sex, smoking history, pathological type, TNM stage, and brain metastasis, showed no statistically significant differences (all *P* > 0.05). However, given the limited sample size, the study has reduced power to detect imbalances; readers are referred to Table 1 for effect sizes to assess potential clinical differences. For instance, numerical differences were observed in the proportion of patients receiving targeted therapy (17.7% vs. 40.0%) and in median CA125 levels (53.00 vs. 24.10). Treatment strategies were diverse and did not differ significantly between groups (all *P* > 0.05). Similarly, serum biomarker levels (CEA, ProGRP, CYFRA21-1, CA125, NSE, SCC) were not significantly different. As detailed in the Methods section, follow-up was defined as the time from the initiation of first-line therapy to the first documented disease progression or the last imaging assessment without progression. As anticipated under this definition, the follow-up duration was significantly shorter in the progression group (median 209 days) than in the non-progression group (median 641 days; *P* < 0.001), reflecting the earlier occurrence of the progression event.

**Table 1:** Clinical characteristics statistics

Characteristics	Progression (*N* = 17)	Non-progression (*N* = 15)	*P*
Age years (Mean ± SD)	57.06 ± 9.81	55.67 ± 12.02	0.717
Sex (%)			> 0.999
Male	8 (47.06)	7 (46.67)	
Female	9 (52.94)	8 (53.33)	
Smoking _Yes (%)	5 (29.41)	3 (20.00)	0.838
Pathological type (%)			> 0.999
Squamous carcinoma	3 (17.65)	3 (20.00)	
Adenocarcinoma	13 (76.47)	12 (80.00)	
Others	1 (5.88)	0 (0.00)	
TNM Stage (%)			
Stage IIIB	2 (11.76)	3 (20.00)	0.879
Stage IVA	5 (29.42)	6 (40.00)	0.529
Stage IVB	10 (58.82)	6 (40.00)	0.288
Brain metastasis (%)	5 (29.41)	3 (20.00)	0.838
Treatment (%)			
Immuno-&chemotherapy	6 (35.29)	6 (40.00)	0.784
Chemotherapy	8 (47.06)	3 (20.00)	0.217
Targeted therapy	3 (17.65)	6 (40.00)	0.269
Osimertinib	2 (11.76)	3 (20.00)	0.879
Alectinib	1 (5.89)	2 (13.33)	0.909
Pralsetinib	0 (0.00)	1 (6.67)	0.469
CEA	4.29 (2.77, 15.87)	3.09 (1.60, 8.60)	0.386
ProGRP	42.20 ± 20.54	40.72 ± 19.21	0.833
CYFRA21-1	7.02 (2.68, 13.12)	6.35 (3.51, 13.54)	0.914
CA125	53.00 (14.60, 172.65)	24.10 (13.40, 33.00)	0.255
NSE	14.69 ± 4.18	17.96 ± 9.72	0.240
SCC	1.03 (0.82, 1.38)	0.91 (0.68, 1.56)	0.481
Follow-up duration (days)			< 0.001*
Median (mean)	209 (268.94)	641 (628.13)	

SD = population standard deviation. For the progression group, follow-up time refers to the interval between the date of diagnosis and the date progression is detected. For the non-progression group, follow-up time refers to the interval between the date of diagnosis and the last day of follow-up. An asterisk (*) indicates a P-value less than 0.05, denoting statistical significance

Table 2 compares conventional static PET/CT metabolic features between the progression and non-progression groups. In these descriptive comparisons, no statistically differences were observed in tumor SUV parameters, bone marrow SUV parameters, or volumetric parameters including MTV and TLG (all *P* > 0.05).

**Table 2:** Comparison of metabolic features between two groups

Metabolic features (Mean ± SD)	Progression (*N* = 17)	Non-progression (*N* = 15)	*P*
T_SUVmax	12.71 ± 4.70	12.43 ± 5.55	0.878
T_SUVmean	7.47 ± 2.70	7.43 ± 3.63	0.966
T_SUVmin	5.10 ± 1.88	4.98 ± 2.22	0.870
BM_SUVmax	2.12 ± 0.47	2.36 ± 2.17	0.672
BM_SUVmean	1.60 ± 0.34	1.57 ± 1.00	0.924
BM_SUVmin	0.98 ± 0.23	0.85 ± 0.35	0.230
MTV	16.37 (7.00, 38.39)	24.95 (3.27, 41.86)	0.885
TLG	172.25 ± 131.85	260.28 ± 274.36	0.269

SD = population standard deviation, BM = bone marrow, T = tumor

### Feature selection and cox model construction

A comprehensive analysis was performed on 47 candidate features, encompassing 11 clinical characteristics (Table 1), 8 conventional metabolic features (Table 2), and 28 TAC features (14 from the tumor and 14 from the bone marrow). Following stable feature selection via the bootstrap-LASSO-Cox procedure (1000 resamples), four imaging features were consistently retained (selection frequency > 50%). These features (detailed in Table 3) were incorporated into a final multivariate Cox proportional hazards model. Within this model, the bone marrow TAC feature BM_C_f__Slope_10−30_ (BCS) demonstrated the strongest and most statistically significant association with progression risk (Hazard Ratio [HR] = 0.10, 95% Confidence Interval [CI]: 0.03 to 0.35, *P* < 0.001). The likelihood ratio test confirmed that the fitted model provided a significantly better fit than the null model (χ² = 15.32, *P* = 0.004). The model’s discriminative performance, assessed by the optimism-corrected Harrell’s C-index via bootstrapping, was 0.77 (95% CI: 0.68 to 0.86).

**Table 3:** Multivariate Cox Regression Analysis

Variable	SF (%)	Coef (95% CI)	HR (95% CI)	*P*
BM_C_f__Slope_10−30_	92.3	-2.30 (-3.51, -1.05)	0.10 (0.03, 0.35)	<0.001*
BM_C_f__AUC_T_	73.6	0.67 (-0.23, 1.57)	1.95 (0.80, 4.79)	0.145
BM_C_f__Slope_max−60_	61.8	0.09 (-0.13, 0.32)	1.10 (0.88, 1.38)	0.406
T_C_f__TAC_max_	78.4	-0.78 (-2.12, 0.55)	0.46 (0.12, 1.73)	0.250

SF = selection frequency, HR = hazard ratio, CI = confidence interval, BM = bone marrow, T = tumor. An asterisk (*) indicates a P-value less than 0.05, denoting statistical significance

### Development of a simplified prognostic tool and biological interpretation

To translate the multivariable model into a parsimonious and clinically practical tool, we sought to identify a single, robust imaging biomarker. Among all candidates, the bone marrow free-component slope between 10 and 30 min post-injection (BCS) demonstrated superior stability, as evidenced by its highest frequency of inclusion across all bootstrap resamples. Therefore, BCS was selected for further evaluation.

To address whether patients destined for different first-line therapies had systematically different pre-treatment BCS values, we compared BCS across the intended treatment modalities. This analysis revealed no significant differences (Supplemental Table 1), supporting that the prognostic value of BCS is independent of the subsequent treatment category in this cohort.

To assess whether BCS measured in non‑involved vertebrae was confounded by a systemic effect from distant bone metastases, we compared BCS values in patients with (*N* = 12) and without (*N* = 20) bone metastases elsewhere (Supplementary Material 2). No statistically significant difference was observed (*P* = 0.284, Mann‑Whitney U test; mean difference − 0.000725, 95% CI − 0.003087 to 0.001638). This finding suggests that the presence of distant osseous metastases does not substantially alter the baseline bone marrow metabolic phenotype in non‑involved vertebral bodies within our cohort.

**Fig. 3:**
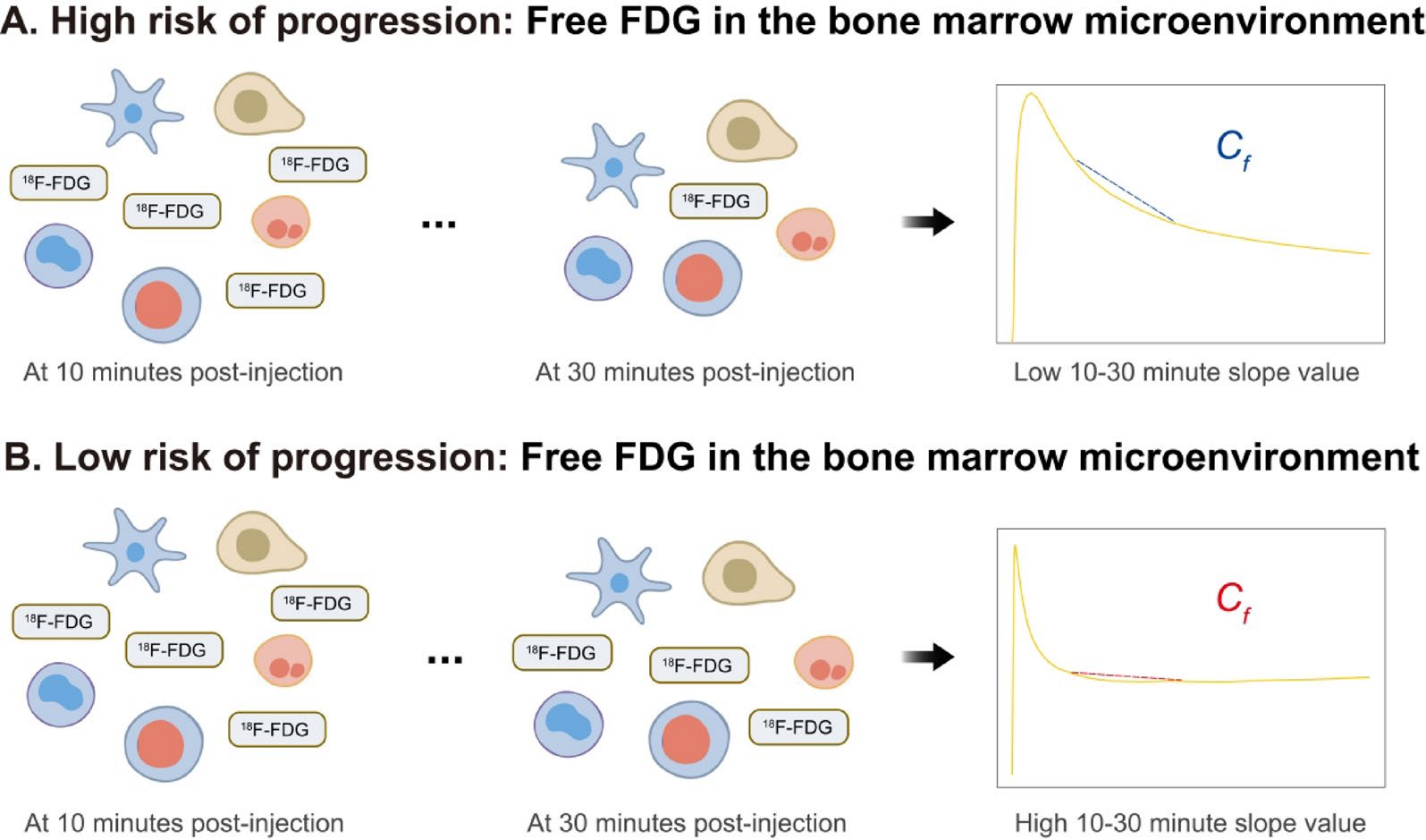
Analysis of free FDG in the bone marrow microenvironment and its association with risk of progression. (**A**) High risk of progression: At 10 min post-injection, free ^18^F-FDG is distributed in the interstitial space surrounding various cell types within the bone marrow. By 30 min post-injection, these levels show a marked decrease. This rapid decline is reflected in a lower (more negative) slope value of the bone marrow free FDG time-activity curve between the 10- and 30-minute time points. (**B**) Low risk of progression: Similarly, free ^18^F-FDG is present in the interstitial space at 10 min post-injection. However, its decrease by 30 min post-injection is more gradual. This slower clearance corresponds to a higher (less negative) slope value on the time-activity curve for the same interval. The difference in the slope of free FDG clearance between the two groups provides insight into the risk of disease progression

Physiologically, BCS quantifies the rate of decline in the model-derived free-fluorodeoxyglucose time-activity curve within the bone marrow. In our initial analysis, a more negative BCS value (steeper decline) was strongly associated with a higher risk of disease progression. This relationship is illustrated in Fig. 3: a steeper decline (more negative BCS) indicates rapid tracer washout and correlates with higher risk (Fig. 3A), whereas a shallower decline (less negative BCS) reflects gradual clearance and correlates with lower risk (Fig. 3B).

To directly assess the inherent prognostic value of BCS independent of model refitting, we subsequently evaluated its predictive performance using time-dependent ROC analysis.

### Risk stratification

**Fig. 4:**
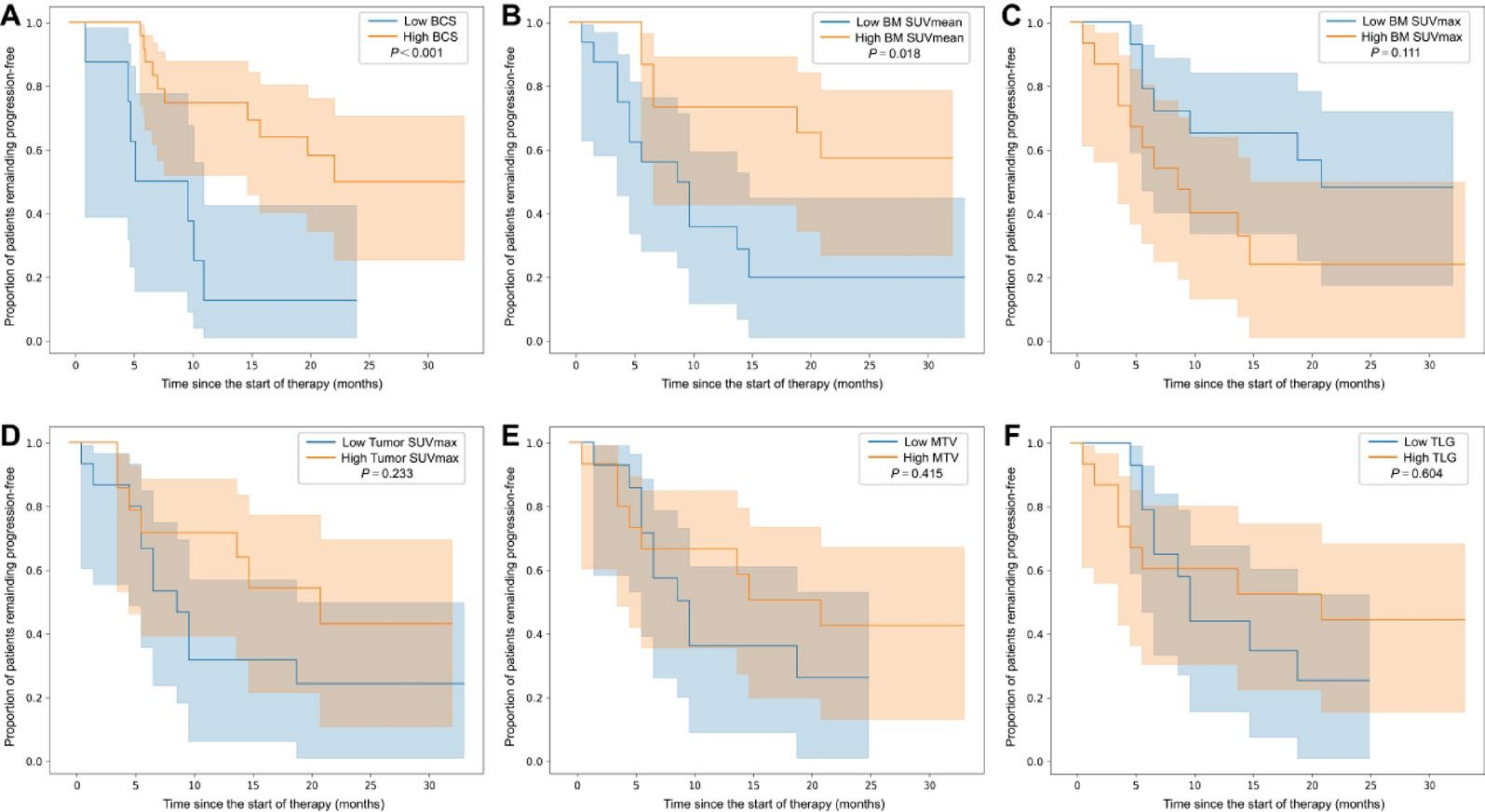
Kaplan-Meier survival curves for progression-free survival stratified by BCS cutoff value. The optimal cutoff value of BCS (− 0.01119) was determined using the log-rank test, and patients were divided into high- and low-risk groups accordingly; the curves show significantly different progression-free survival (panel A). Additional panels depict stratification by BM SUVmean (B, *P* = 0.018), BM SUVmax (C, *P* = 0.111), tumor SUVmax (D, *P* = 0.233), metabolic tumor volume (E, *P* = 0.415), and total lesion glycolysis (F, *P* = 0.604), each dichotomized by the optimal log-rank cutoff; shaded areas indicate 95% confidence intervals, orange and blue denote high and low groups, and time is shown in months since therapy start. BM = bone marrow

**Fig. 5:**
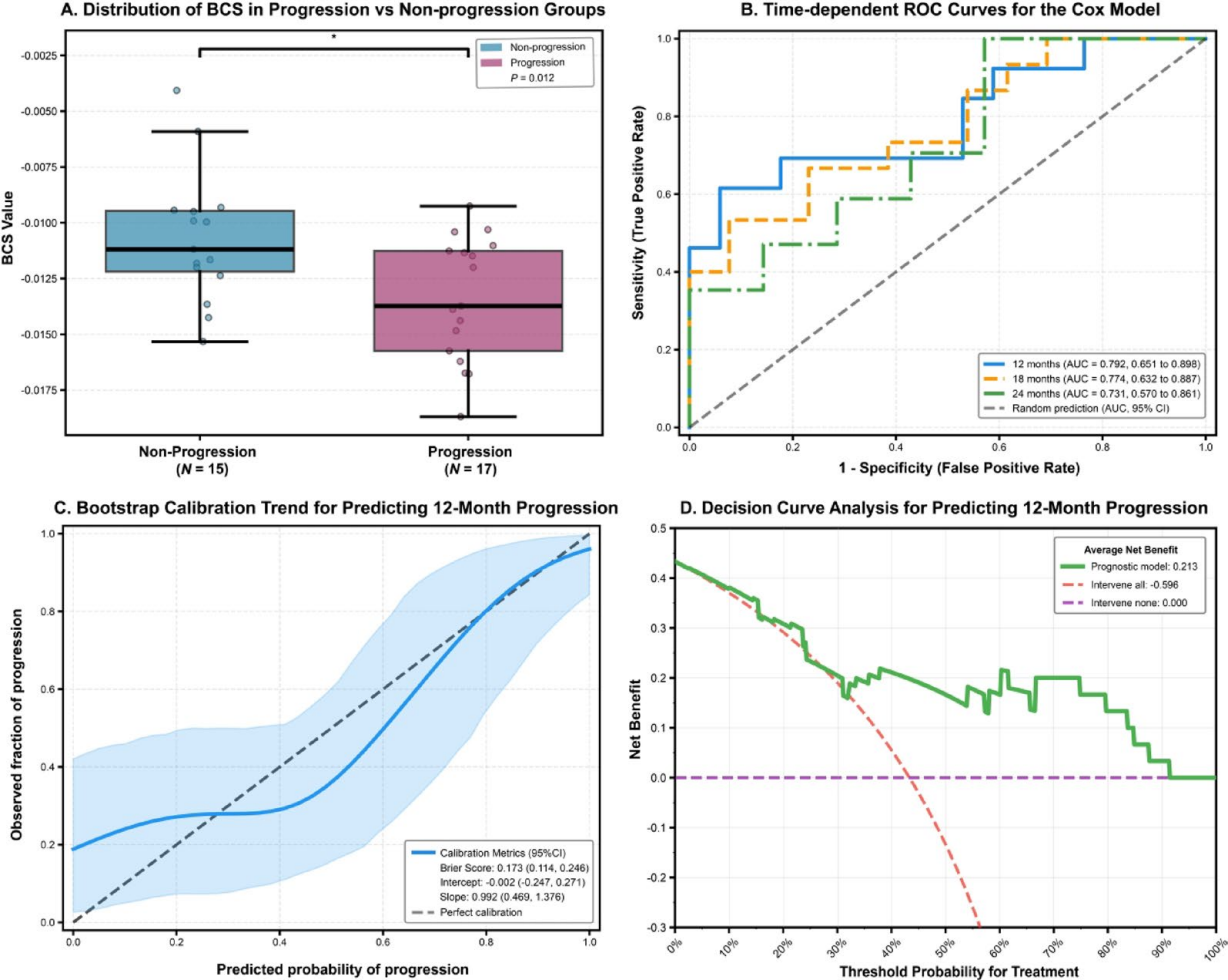
Prognostic performance and clinical utility of the BCS model for progression prediction. (A) Distribution of the BCS values between the non-progression group (N = 15) and the progression group (N = 17). Patients who experienced progression exhibited significantly lower BCS values compared with those without progression (P = 0.012). (B) Time-dependent receiver operating characteristic (ROC) curves evaluating the discriminative ability of the BCS model for predicting progression at 12, 18, and 24 months. The corresponding areas under the curve (AUCs) were 0.792, 0.774, and 0.731, respectively, indicating good temporal predictive performance. (C) Bootstrap calibration curve for predicting 12-month progression, demonstrating the agreement between predicted probabilities and observed outcomes. The shaded area represents the 95% confidence interval. Model calibration metrics included a Brier score of 0.173, an intercept of − 0.002, and a slope of 0.992, suggesting good calibration. (D) Decision curve analysis for predicting 12-month progression. The BCS model provided a net benefit of 0.213 across a wide range of clinically relevant threshold probabilities (2.5% to 91.3%), outperforming both the “intervene-all” and “intervene-none” strategies.

The optimal prognostic cut-off value for BCS was determined to be -0.01119 using the Log-rank test. Patients were stratified into high-risk (BCS ≤ -0.01119) and low-risk (BCS > -0.01119) groups. Kaplan-Meier analysis confirmed that BCS strongly stratified PFS, with a clear and sustained separation of curves from early time points (Log-rank *P* < 0.001; Fig. 4A). The BCS values were also significantly lower in patients who experienced progression compared to those who did not (*P* = 0.012; Fig. 5A).

As an exploratory analysis, conventional metabolic metrics were also examined. Only bone marrow SUVmean showed a significant association with PFS after dichotomization using the same data-driven method (Log-rank *P* = 0.018; Fig. 4B). However, its prognostic value appeared to be highly dependent on the dichotomization itself. When treated as a continuous variable in a univariable Cox model, bone marrow SUVmean was not significantly associated with progression risk (HR = 1.14; 95% CI: 0.66 to 1.96; *P* = 0.637). This lack of consistency across analytical approaches suggests that the significant Log-rank P value may largely stem from the data-driven optimization of the single cut-point, rather than from a stable, underlying association. Consequently, this particular finding should be interpreted with considerable caution.

### Prognostic performance of the simplified BCS prognostic model

The performance of the final single-feature (BCS) risk stratification model was evaluated. To quantify temporal discrimination, time-dependent ROC analysis was performed at pre-specified horizons of 12, 18, and 24 months using a cumulative definition, which accounts for censoring. The AUC at each horizon, along with 95% CIs estimated via bootstrap, was as follows: 0.792 (95% CI: 0.651 to 0.898) at 12 months, 0.774 (95% CI: 0.632 to 0.887) at 18 months, and 0.731 (95% CI: 0.570 to 0.861) at 24 months (Fig. 5B). Internal validation using bootstrap optimism correction was applied to assess calibration at 12 months. The optimism-corrected calibration curve showed agreement between predicted and observed event rates (Fig. 5C). The calibration intercept and slope estimates were close to their ideal values, although CIs were wide, consistent with limited sample size. Finally, DCA at 12 months was performed to explore the model’s potential utility across a spectrum of threshold probabilities. The DCA demonstrated that, across a wide range of thresholds, the BCS model provided higher net benefit than both the “treat-all” and “treat-none” strategies (Fig. 5D).

### Illustrative Cases

**Fig. 6:**
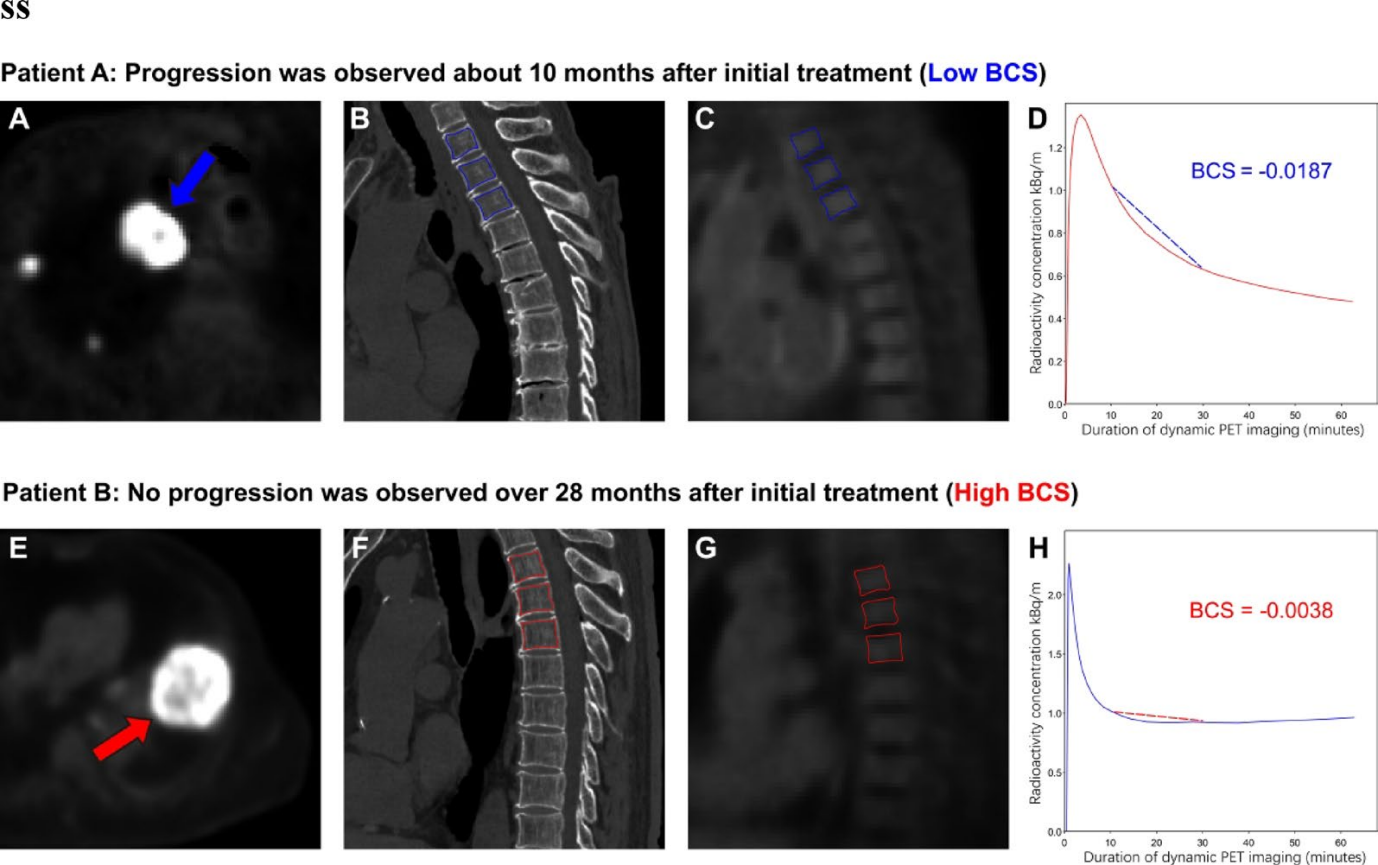
Representative ^18^F-FDG PET/CT cases illustrating BCS in patients with different outcomes. Patient A (Low BCS; progression about 10 months after initial treatment): 74-year-old man with right-lung squamous cell carcinoma and multiple metastases. (**A**) Axial PET shows a hypermetabolic pulmonary lesion (blue arrow). (**B**) Sagittal CT with bone marrow ROIs at T3–T5 (blue boxes). (**C**) Matched sagittal PET with the same ROIs. **D**) TAC of the bone marrow component $\:{C}_{f}$; the dashed segment indicates the slope used to compute BCS (BCS = − 0.0187). Patient B (High BCS; no progression for over 28 months): 69-year-old man with left-lung adenocarcinoma and widespread metastases. (**E**) Axial PET shows a hypermetabolic pulmonary lesion (red arrow). (**F**) Sagittal CT with ROIs at T3–T5 (red boxes). (**G**) Matched sagittal PET with the same ROIs. (H) TAC of the bone marrow component $\:{C}_{f}$; the dashed segment indicates the slope used to compute BCS (BCS = − 0.0038).

Figure 6 presents two illustrative cases, selected as representative examples from their respective risk groups. The high-risk patient (BCS = -0.0187) experienced disease progression, confirmed on a scheduled contrast-enhanced CT scan at approximately 10 months after initiation of first-line therapy. In contrast, the low-risk patient (BCS = -0.0038) showed no evidence of progression on the last scheduled contrast-enhanced CT assessment, performed approximately 28 months after treatment initiation; this patient was therefore censored at that time point, consistent with the protocol-defined surveillance schedule.

## Discussion

This study advances our understanding of progression risk stratification in advanced NSCLC patients who achieve PR following first-line therapy. More specifically, it makes a dual contribution: it introduces a novel, physiology-informed imaging biomarker, and it shifts the prognostic paradigm from a tumor-centric to a systemic, host-focused perspective. We demonstrate that heterogeneity in bone marrow glucose kinetics, quantified through dynamic ^18^F-FDG PET imaging, is associated with disease progression risk and serves as a promising biomarker reflecting underlying host biology.

Our central finding reveals that the slope of the free ^18^F-FDG component within the bone marrow microenvironment during the critical 10–30 min post-injection window, termed BCS, stratifies PR patients into prognostically distinct groups with striking clarity. Patients exhibiting lower BCS values, indicative of a steeper decline in free tracer concentration within the bone marrow, faced a dramatically elevated risk of progression. The predictive value of BCS was robustly validated: a single-feature model incorporating BCS demonstrated strong and consistent discriminatory power over time (AUCs: 0.792, 0.734, and 0.731 at 12, 18, and 24 months, respectively), excellent calibration, and superior clinical utility in decision curve analysis compared to non-stratified strategies (Fig. 5B-D). This performance starkly contrasts with the lack of predictive value from conventional tumor-centric metrics in our cohort, underscoring that progression risk in this population is governed less by the residual focal tumor burden visible on imaging [24, 25] and more by systemic factors [26]. We propose that the bone marrow acts as a “systemic biosensor”, and its early metabolic kinetics after therapy initiation reveal the host’s capacity for sustaining a durable anti-tumor response. This paradigm shift necessitates biomarkers capable of probing this systemic dimension.

Methodologically, our approach represents an evolution beyond prior dynamic PET investigations. While previous studies have highlighted the potential of kinetic parameters over static SUV [8, 27], they often relied on complex compartmental modeling prone to errors from model misspecification and partial volume effects [28, 29]. Others utilized composite TACs [30, 31], but these amalgamate signals from blood flow, vascular permeability, extracellular diffusion, and cellular phosphorylation, rendering their physiological interpretation ambiguous. Our innovation lies in decomposing the tissue TAC into three physically and physiologically distinct components derived from the two-tissue compartment model framework: vascular ($\:{C}_{b}$), free extracellular ($\:{C}_{f}$), and metabolically trapped ($\:{C}_{m}$) ^18^F-FDG. This decomposition allowed us to isolate and interrogate specific kinetic processes. It was this level of detail that revealed BCS, which captures the retention dynamics of free ^18^F-FDG in the bone marrow, as the paramount predictive feature rather than features derived from the tumor itself or the composite signal. This targeted compartmental analysis provides unprecedented interpretability: BCS directly reflects the kinetics of glucose analogue movement and potential retention within the extracellular space of the bone marrow microenvironment.

Biologically, a low BCS (rapid decline in free FDG) is consistent with a dysfunctional bone marrow niche. This could manifest through several interconnected pathways. It may signify an expansion of immunosuppressive cell populations, such as myeloid-derived suppressor cells, which exhibit altered glucose metabolism and may rapidly consume extracellular glucose or alter interstitial fluid dynamics [32–34]. Alternatively, it could reflect pathological remodeling of the bone marrow stroma, impairing normal metabolite exchange or creating hypoxic pockets that influence tracer kinetics [35, 36]. Critically, the bone marrow is not merely a passive bystander but acts as a systemic “bioreactor”, influencing distant tumor progression through the continuous output of immune cells, cytokines, and pro-angiogenic factors [37]. An altered metabolic signature, as captured by BCS, likely signifies a shift in this output towards a pro-tumorigenic state, fostering immune evasion, angiogenesis, and ultimately, clinical progression. Thus, BCS may be an early, integrative readout of a failing systemic anti-tumor immune response.

The clinical implications are significant and bolstered by a key practical advantage: the derivation of BCS relies solely on dynamic data acquired between 10- and 30-minutes post-injection. This focused 20-minute window drastically reduces the acquisition time compared to traditional dynamic PET protocols often requiring 60 min, addressing a major barrier to clinical implementation related to patient comfort, scanner throughput, and motion artifact potential. Given its strong predictive performance, BCS could offer a practical, non-invasive tool for personalizing post-PR management in future validation. This could enable a risk-adapted management framework: patients identified as high-risk (low BCS) could be enrolled in intensified surveillance protocols and become prime candidates for clinical trials testing novel consolidation strategies. Conversely, low-risk patients (high BCS) might be considered for standard follow-up or even de-escalation strategies. This stratification, informed by a biologically-grounded systemic metric, moves beyond reactive management towards pre-emptive and precision care.

Several limitations of this study should be acknowledged. First, this study has inherent limitations in external validity due to its single-center design and relatively small sample size. While the BCS threshold of -0.01119 demonstrated robust prognostic value within our cohort under standardized conditions, it is critical to note that this and all other data-driven cut-off analyses in this study are exploratory in nature. Consequently, the generalizability and reproducibility of such thresholds require confirmation. Variability in imaging protocols and reconstruction algorithms across centers may influence this quantitative threshold. Therefore, future prospective, multi-center studies with larger cohorts and harmonized technical standards are essential to independently verify the observed association of BCS with clinical outcomes. Second, the longer duration of dynamic PET compared to conventional PET scanning may challenge patients with poor conditions and increase motion artifacts [38]. However, limiting image acquisition to the 10–30 min post-injection window can effectively reduce scanning time. Third, the cohort exhibited heterogeneity in treatment regimens. Although baseline BCS values were comparable across treatment groups (*P* = 0.634), we acknowledge that the lack of statistical adjustment for treatment modality in our primary survival analysis represents a study limitation. Treatment type remains a potential residual confounder in the observed association between BCS and PFS, particularly given the small sample size and heterogeneous regimens. Furthermore, this study has biological correlative limitations. Without immunohistochemical staining of tumor biopsies or bone marrow samples for analysis, we could not profile the metabolic or immune microenvironment. Consequently, we could not correlate the bone marrow glucose kinetic signature with local tissue phenotypes or elucidate how the observed metabolic heterogeneity influences immune responses and disease progression. Future prospective studies with systematic tissue and bone marrow molecular profiling are essential to establish these mechanistic links.

## Conclusions

In conclusion, this study establishes that bone marrow glucose kinetic heterogeneity, quantified specifically by the BCS parameter derived from dynamic ^18^F-FDG PET/CT imaging, is a novel imaging biomarker associated with progression-free survival in advanced NSCLC patients achieving partial response. More importantly, it suggests that the biological context of treatment response may be redefined by identifying the host’s bone marrow metabolic state as a potential critical determinant of long-term outcome. This biomarker may provide a practical tool for early risk stratification, which could enable a shift from uniform follow-up to personalized, risk-adapted management. Furthermore, it paves the way for future trials exploring systemic immunity-targeted interventions for high-risk patients. Validation in larger cohorts and elucidation of the underlying biological mechanisms are essential next steps toward integrating this biomarker into precision oncology paradigms.

## Electronic Supplementary Material

Supplementary Material 1

Supplementary Material 2

Supplementary Material 3

## Data Availability

The datasets generated and/or analysed during the current study are not publicly available due to patient privacy and ethical restrictions but are available from the corresponding author on reasonable request. Data access is subject to approval by the institutional ethics committee and a signed data use agreement.

## Abbreviations

NSCLC: Non-small cell lung cancer
PR: Partial response
TAC: Time-Activity Curve
PFS: Progression-free survival
ROI: Region of interest
SUV: Standardized uptake value
MTV: Metabolic tumor volume
TLG: Total lesion glycolysis
AUC: Area under the receiver operating characteristic curve
ROC: Receiver operating characteristic
DCA: Decision curve analysis
BCS: BM_C_f__Slope_10−30_
KM: Kaplan-Meier
