# Supplementary material for: From imaging heterogeneity to clinical decision: a novel imaging biomarker based on bone marrow kinetics for advanced NSCLC patients in partial response
Source: EJNMMI Res. 2026 May 21;16:83. doi: 10.1186/s13550-026-01440-w (PMC13194849; doi:10.1186/s13550-026-01440-w)
Supplement: Supplementary file 2 — Supplementary Material 2 [file 13550_2026_1440_MOESM2_ESM.docx]

**Supplemental Table 1** Comparison of BCS among different treatment regimens

| Treatment Group | N | Mean ± SD | P-value for Pairwise Comparisons | | |
| --- | --- | --- | --- | --- | --- |
|  |  |  | vs. A | vs. B | vs. C |
| A. Immuno-Chemotherapy | 12 | -0.0116 ± 0.0037 | - | > 0.999 | > 0.999 |
| B. Chemotherapy | 11 | -0.0129 ± 0.0022 | > 0.999 | - | > 0.999 |
| C. Targeted Therapy | 9 | -0.0120 ± 0.0034 | > 0.999 | > 0.999 | - |

Data are presented as mean ± standard deviation. N represents the number of patients in each group. Group designations: A, Immuno-Chemotherapy; B, Chemotherapy; C, Targeted Therapy. One-way ANOVA revealed no statistically significant difference among the three groups (*F* = 0.464, *P* = 0.634). Post-hoc pairwise comparisons using t-test with Bonferroni correction also showed no significant differences between any two groups (all adjusted *P* > 0.999).
