# Supplementary material for: From imaging heterogeneity to clinical decision: a novel imaging biomarker based on bone marrow kinetics for advanced NSCLC patients in partial response
Source: EJNMMI Res. 2026 May 21;16:83. doi: 10.1186/s13550-026-01440-w (PMC13194849; doi:10.1186/s13550-026-01440-w)
Supplement: Supplementary file 3 — Supplementary Material 3 [file 13550_2026_1440_MOESM3_ESM.docx]

**Supplementary Material 2. Comparison of BCS by bone metastasis status**

**Descriptive statistics:**

With bone metastasis (N = 12): Mean ± SD = -0.01259 ± 0.00417; Median (IQR) = -0.01381 (-0.01543 to -0.01071)

Without bone metastasis (N = 20): Mean ± SD = -0.01187 ± 0.00240; Median (IQR) = -0.01142 (-0.01209 to -0.01022)

**Normality testing (Shapiro-Wilk test):**

With bone metastasis: *W* = 0.862, *P* = 0.051 (normal distribution)

Without bone metastasis: *W* = 0.850, *P* = 0.005* (non-normal distribution)

Between-group comparison:

Test method: Mann-Whitney U test (nonparametric)

Test statistic (U): *U* = 92.000

p-value (two-tailed): *P* = 0.284

Effect size (r): *r* = 0.189 (small effect)

Mean difference (with metastasis - without metastasis): -0.000725

95% confidence interval: -0.003087 to 0.001638

Note: *The group without bone metastasis violated the normality assumption (*P* < 0.05), therefore the nonparametric Mann-Whitney U test was used for between-group comparison. Effect size r was calculated as r = Z/√N. The 95% confidence interval for the mean difference was calculated using bootstrap method (1000 iterations). SD, standard deviation; IQR, interquartile range.
